# Enteric Pathogens in Humans, Domesticated Animals, and Drinking Water in a Low-Income Urban Area of Nairobi, Kenya

**DOI:** 10.1021/acs.est.4c10041

**Published:** 2024-11-26

**Authors:** Sean W. Daly, Benard Chieng, Sylvie Araka, John Mboya, Christine Imali, Jenna M. Swarthout, Sammy M. Njenga, Amy J. Pickering, Angela R. Harris

**Affiliations:** † Department of Civil, Construction, and Environmental Engineering, North Carolina State University, Fitts-Woolard Hall, 915 Partners Way, Rm 3250, Raleigh, North Carolina 27695, United States; ‡ Kenya Medical Research Institute, Nairobi 00100, Kenya; § Department of Civil and Environmental Engineering, University of California, Berkeley, Berkeley, California 94720, United States; ∥ Innovations for Poverty Action, Nairobi 00100, Kenya; ⊥ Civil and Environmental Engineering, Tufts University, Medford, Massachusetts 02155, United States; # Chan Zuckerberg Biohub, San Francisco, California 94158, United States

**Keywords:** zoonotic pathogen, microbial source tracking, TaqMan Array Card, host−pathogen relationship, drinking water quality, low- and middle-income country

## Abstract

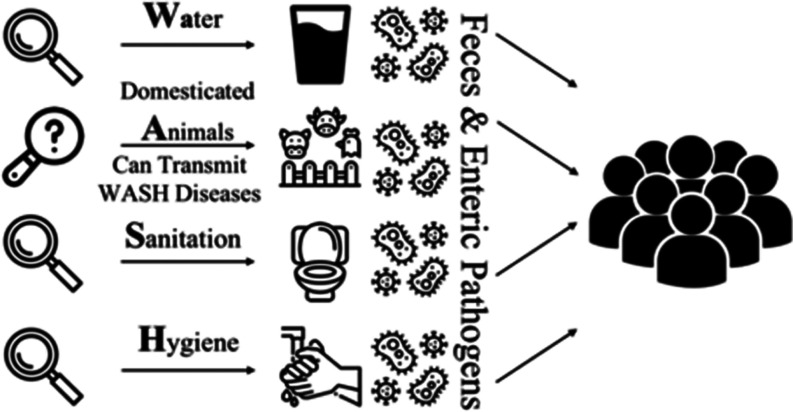

To explore the sources of and associated risks with drinking water contamination in low-income, densely populated urban areas, we collected human feces, domesticated animal feces, and source and stored drinking water samples in Nairobi, Kenya in 2019; and analyzed them using microbial source tracking (MST) and enteric pathogen TaqMan Array Cards (TACs). We established host–pathogen relationships in this setting, including detecting *Shigella* and Norovirus—which are typically associated with humans—in dog feces. We evaluated stored and source drinking water quality using indicator *Escherichia coli* (*E. coli*), MST markers, and TACs, detecting pathogen targets in drinking water that were also detected in specific animal feces. This work highlights the need for further evaluation of host–pathogen relationships and the directionality of pathogen transmission to prevent the disease burden associated with unsafe drinking water and domestic animal ownership.

## Abstract 2

This manuscript provides evidence of understudied zoonotic host−pathogen relationships and identifies methods for identifying potential zoonotic pathogen transmission to humans.

## Introduction

Diarrheal disease is a leading cause of death worldwide in children under 5 years, with most cases occurring in low- and middle-income countries (LMICs).^1^ Infrastructure insufficiencies in these areas can promote the spread of disease-causing pathogens,^2^ and insufficient water, sanitation, and hygiene (WASH) conditions are associated with 60% of total diarrheal disease deaths.^3^ Drinking water in particular has been identified as a dominant pathway of transmission of enteric pathogens,^4,5^ with Julian (2016)^6^ identifying enterotoxigenic *Escherichia coli* (*E. coli*), enteropathogenic *E. coli*, *Shigella* spp., *Cryptosporidium* spp., rotavirus, and norovirus as important etiologies of diarrheal disease in LMICs. These and other pathogens can spread to humans through both improperly managed human feces and from animal feces to humans, which is an area of particular concern for LMICs.^7^

Despite rapid urban growth in these regions, animal husbandry remains a common and valuable economic resource for members of the population.^8^ Domesticated animal ownership has already been associated with pathogen presence in the surrounding environment^9^ and negative health outcomes for humans.^10^ It is also common in LMICs for domesticated animals to be kept around or inside the home or living spaces, raising the risk for exposure to animal feces.^8^ Like any environmental contaminant, zoonotic pathogens can be transmitted through WASH pathways such as drinking water.^9^ Proper animal feces management has been identified as a challenge or limitation for many common WASH intervention methods;^11,12^ and neglecting to include animal feces management in sanitation interventions^13,14^ could lead to diminished impacts of WASH intervention campaigns^15^ and persisting negative health outcomes.^9^ Soils, human hands,^16^ meats,^17^ produce,^18^ household items (e.g., children’s toys),^19^ and drinking water^9^ have been identified as pathways by which humans are exposed to animal feces or zoonotic pathogens. Animal husbandry more generally has been associated with diarrheal disease in humans.^20^ Beyond diarrhea, there is evidence that exposure to domesticated animals and their feces can lead to environmental enteric dysfunction (a condition resulting in growth and cognitive impairment), trachoma, and increased risk of infection with soil-transmitted helminths.^9^ However, there is uncertainty surrounding which animal hosts are most likely harboring or transmitting certain pathogens. Many pathogens are carried by a variety of hosts, including humans and domesticated animals.^21,22^ While some diseases are zoonotic in origin (e.g., rabies), this uncertainty contributes to the gap in knowledge surrounding identifying the animal source of pathogens or the direction of animal-human transmission pathways. Increasing surveillance and developing tools for enhancing understanding of the animal-human disease interface have been identified as crucial steps toward the One Health framework for managing zoonotic diseases. Understanding what animal hosts are most likely carrying and transmitting certain pathogens is critical for effective management of human and animal infectious disease, and for achieving the One Health objective for preventing, detecting, and responding to disease threats.^23^

Historically, fecal indicator or model organisms such as *E. coli* have been used as a proxy organism to suggest the presence of pathogens in drinking water or other environmental samples.^24^ In fact, the World Health Organization Guidelines for Drinking Water Quality do not necessarily indicate that water must be pathogen free, only that *E. coli* or thermotolerant coliforms (TTC) must not be detected in a 100 mL sample of water.^25^ Indicator organisms such as *E. coli* have been successful regulatory tools for assessing water quality, as there is no comprehensive method for testing for all pathogens, and some cannot be cultured using traditional methods.^24^ However, there is some evidence that coliform indicators may not be sufficient for modeling enteric viruses and protozoan pathogens,^26^ and that some pathogens with high infectivity such as *Shigella* spp., *Cryptosporidium* spp., rotavirus, and norovirus could sufficiently contaminate drinking water to cause disease even when *E. coli* is not detected in 100 mL of water.^6^

The rise of molecular, polymerase chain reaction (PCR) methods for detecting microorganisms in the environment offers an alternative to indicator methods. Amplifying specific gene targets using PCR provides a reliable tool for the detection of various infectious agents both in hosts and in the environment, and has been used for evaluating the microbial quality of drinking water and other environmental samples.^24^ Molecular PCR methods can provide separate information from indicator organisms, such as the source of fecal contamination in the environment. *Bacteroidales* gene markers can be used with quantitative PCR (qPCR) amplification to detect gene markers specific to the feces of certain animal hosts, an approach known as microbial source tracking (MST). Among many others, some common MST markers used in LMIC settings to evaluate zoonotic contamination of drinking water are HF183, Rum2Bac, and Avian GFD, used to identify human, ruminant, and avian feces, respectively.^16,27^ The performance of such assays, meaning the level to which they are sensitive and specific to their target host (i.e., the HF183 marker’s sensitivity for detecting human feces), can vary by geography. Therefore, on-site validation is commonly done for these MST assays.^16,27−30^ TaqMan Array Cards (TACs) are another molecular detection platform, capable of simultaneous real-time PCR (RT-PCR) amplification of up to 48 gene targets.^31^ TACs have been successfully used to detect large panels of pathogen gene targets in a variety of samples, including environmental samples such as water.^32,33^ The simultaneous amplification of multiple targets, as opposed to traditional, single-target PCR protocols, provides a powerful investigatory tool, given the wide range of relevant pathogens that exist in and are transmitted through environmental pathways. Using TACs to detect specific pathogen targets could identify common pathogens in the feces of particular animal hosts, and using TACs to evaluate drinking water could alleviate the limitations of using nonspecific measures such as indicator organisms.

In this study, we evaluated the presence and potential sources of enteric pathogens in source and stored drinking water in a densely populated, low-income urban area of Kenya. We assessed the sensitivity and specificity of three microbial source tracking qPCR assays in this setting, then applied these assays to source and stored drinking water samples to identify the source of fecal contamination in drinking water. Additionally, we used a TaqMan Array Card PCR platform to investigate the presence of multiple enteric pathogens in the collected feces samples to address the uncertainty surrounding host–pathogen relationships in this setting. These molecular methods, coupled with indicator *E. coli* enumeration, were used to evaluate the quality of drinking water in this community, and to investigate the strengths of molecular methods compared to traditional indicator methods.

## Methods

### Sample Collection

Sample collection was conducted in the Dagoretti South subcounty of Nairobi, Kenya in 2019. These samples were collected as a part of a larger campaign to investigate the use of household environmental sampling for surveillance of soil-transmitted helminths, and the impact of animal husbandry (specifically poultry) on fecal bacteria contamination.^34,35^ Inclusion criteria for household selection included requiring at least one child <5 years of age living in the household, and 47 households were identified and enrolled to participate in the study. Enumerators underwent a 5-day training session prior to sample collection to ensure compliance with informed consent procedures, understanding of survey protocols and tools, and training for sample collection and handling. A stored drinking water sample was collected at each household (*n* = 46), and the number of source water (*n* = 13), human fecal (*n* = 22), and domesticated animal fecal (*n* = 111) samples collected was determined based on availability. Household stored drinking water was collected by pipetting 350 mL water from the bottom of storage containers into sterile plastic sampling bags using sterile serological disposable pipettes. Source drinking water was collected by aliquoting 350 mL water from the source directly into sterile plastic sampling bags. The samples were comprised of 25 piped water samples, 29 borehole water samples, and 5 samples collected from water tanker trucks. The storage containers used for household water storage included jerry cans, plastic water bottles, and other plastic containers (e.g., buckets and jugs). Of the 59 total collected water samples, 54 contained sodium thiosulfate to neutralize residual chlorine,^36^ though free chlorine was not detected in the majority (>91%) of drinking water samples collected in the area.^34^ Domesticated animal feces was collected from chickens (*n* = 26), cows (*n* = 24), dogs (*n* = 20), ducks (*n* = 20), and goats (*n* = 21). Trained field staff identified and collected fresh animal feces into 50 mL centrifuge tubes using a sterile collection spoon and avoiding soil contamination. Human feces was collected, from both adults (18+ years of age) and children (0–15 years of age), by providing household primary caretakers a stool collection kit, and instructing caretakers to collect feces the morning of, or night before, follow-up/collection visits. Households were visited up to three times to achieve successful stool collection. Caretakers were instructed to collect feces on aluminum foil, then, using sterile gloves and scoops, transfer the feces to a 50 mL sterile feces collection tube for collection. Field blanks for water samples were generated by providing field staff a sterile bottle of 200 mL of water to pour into a sterile plastic sampling bags during field sampling. All samples were transferred same-day on ice in a cooler to the field lab for processing.

Following collection and transfer to the Kenyan field lab, 100 mL of each drinking water sample was vacuum filtered onto Millipore 0.45-μm HA membrane filters and transferred using ethanol and flame sterilized forceps into PowerBead Pro Tubes (Qiagen, Valencia, CA) for later nucleic acid extraction. Laboratory blanks for water samples were generated once per day, by rinsing the sides of the membrane filtration funnel with deionized water, without adding a sample. To enumerate *E. coli* in drinking water samples, 100 mL of undiluted water was membrane filtered and then incubated on Tryptone Bile X-glucuronide (TBX) agar plates for 18 h at 44 °C. If the result was too numerous to count, it was substituted with 500 CFU per 100 mL for statistical analysis, as no sample remained for subsequent dilution and reculture. For each feces sample, 0.25 g was weighed out using sterile spoons and aluminum weigh trays, and then transferred to a PowerBead Bead Tube (Qiagen, Valencia, CA) for later nucleic acid extraction. Following transfer to the appropriate tubes, samples were stored at −80 °C. Samples were then transported on dry ice from Kenya to North Carolina State University laboratories, with appropriate United States Department of Agriculture and Centers for Disease Control and Prevention permits, for nucleic acid extraction and PCR analysis.

### Nucleic Acid Extraction

DNA and RNA were extracted using the commercial RNeasy PowerMicrobiome kit (Qiagen, Valencia, CA) for feces samples and the DNeasy PowerSoil Pro kit (Qiagen, Valencia, CA) for drinking water samples. Different kits were used as the initial project scope included investigating just DNA targets, and extraction kits were switched to include RNA capture for fecal samples. In order to capture DNA, the RNA isolation steps in the RNeasy PowerMicrobiome kit protocol were excluded. DNA was extracted from filtered drinking water samples by following the DNeasy PowerSoil Pro kit protocol for environmental samples. Prior to extraction, domesticated animal fecal samples were spiked with 10 μL of the TaqMan Universal DNA Spike-In Control (Qiagen, Valencia, CA), also called the Xeno control. Human feces samples and drinking water samples were not spiked with the Xeno extraction control, as the control was only obtained after extraction of water and human fecal samples. Up to 24 samples were extracted in a batch, with an extraction blank created with each batch of samples. The DNA and RNA concentration in sample extracts were determined using Nanodrop (Thermo Fisher Scientific, Waltham, MA, model ND-1000).

### MST Assay Validation

The MST assay validation procedure was conducted using an established method.^16,27^ The following assays were used for detecting human, ruminant, and avian feces, respectively: TaqMan HF183,^37^ TaqMan Rum2Bac,^38^ and SYBR Avian GFD.^39^ The assays were used in both “target host” (i.e., human specific HF183 assay in human feces) feces and “nontarget host” feces (i.e., HF183 in cow feces). All reaction mixtures, template DNA volumes, and thermocycling parameters can be seen in the Supporting Information. For qPCR comparison, the same number of samples per feces source was desired. With the lowest total of samples collected by source being 20 (dogs, ducks), 20 samples per feces source were analyzed. All collected waters were analyzed with all three MST assays, which included 46 stored water samples, 13 source water samples, 11 field blanks, 11 lab blanks, and 10 extraction blanks. A standard curve was run on each qPCR plate containing concentrations of standard (i.e., DNA target for each assay) ranging from 10^1^ to 10^5^ copies per μL of template. Standards for each assay were MiniGene products obtained from Integrated DNA Technologies, quantified using Nanodrop (Thermo Fisher Scientific, Waltham, MA, model ND-1000). A no template control was also included in each plate. All samples, standards, and controls were run in triplicate on each plate. For each sample, the number of copies of target DNA per μL was calculated using that specific plate’s standard curve, then divided by the concentration of DNA (ng/μL) in the same samples, and the concentrations of the MST targets were reported per nanogram of extracted DNA.^16^ Samples were considered positive if two or three of the triplicate reactions successfully amplified the target DNA sequence. If just one, or none, of the triplicate reactions amplified, the sample was considered a nondetect. If a sample amplified, but was below 10^1^ copies per μL, it was considered detected but not quantifiable. All qPCR analysis was conducted using a QuantStudio 7 Flex Real-Time PCR System (Thermo Fisher Scientific,Waltham, MA).

### Evaluating Enteric Pathogen Presence with TaqMan Array Cards

We used a gastrointestinal enteric pathogen TaqMan Array Card developed by Thermo Fisher Scientific, specifically the “Gastrointestinal Trial Card, Version 3.” These proprietary cards contain targets, including DNA and RNA, for 43 enteric pathogens (three *E. coli* targets run in duplicate), and two internal controls (see Supporting Information for more details). These cards allow for the detection of 24 bacterial, 13 viral, and 6 protozoan pathogen targets. First, samples were preamplified using TaqPath 1-Step RT-qPCR Master Mix, GC (Thermo Fisher Scientific, Waltham, MA) and a custom designed primer pool. The preamplified samples were then diluted 1:10 in nuclease-free water, and combined with TaqMan Fast Advanced Master Mix, no UNG for the final reaction mixture (see Supporting Information Tables SI1–SI10 for all reaction mixture volumes and thermocycling parameters). This final reaction mixture was added to the TAC for PCR amplification of 8 samples simultaneously using a QuantStudio 7 Flex Real-Time PCR System (Thermo Fisher Scientific, Waltham, MA).

### Data Analysis

For the MST assays, the mean copies of target gene per nanogram of DNA extracted were calculated and the difference between the mean estimates among different feces sources were compared using a *t* test. Following the qPCR analysis, the sensitivity and specificity of each assay to each target feces source was calculated using the following established equations:^16^
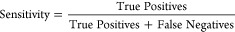
1
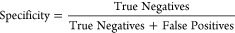
2A commonly used presence/absence or binary baseline of acceptable sensitivity and specificity for microbial source tracking assays is 0.80, or 80%,^27^ and this threshold was used to evaluate whether the MST assays were considered “sensitive” or “specific” in this study context.

To compare the concentration of MST markers and the average number of pathogens detected by feces source using the TACs, a Shapiro-Wilk normality test was first used to determine the frequency distribution of the number of positive pathogen targets by feces source. After identifying that not all distributions were normal or log-normal, a nonparametric Kruskal–Wallis Dunn’s test for multiple comparisons was used. Comparing the proportion of samples containing each individual pathogen between feces sources was conducted using two-proportion Z-tests, where all possible combinations of hosts were compared (i.e., for each pathogen, chicken vs cow, chicken vs dog, etc.). Determining correlations between source and stored water and contamination, and between different types of drinking water contamination (e.g., *E. coli* and adenovirus) was conducted using Fisher’s Exact tests. All tests were conducted using an α confidence level of 0.05. All analyses were conducted using the “stats” and “FSA” packages in R version 4.0.5. Methods and results for investigating and resolving PCR inhibition were conducted using established methods^16^ and are described in the Supporting Information.

## Results

### MST Assay Validation

**Figure 1:**
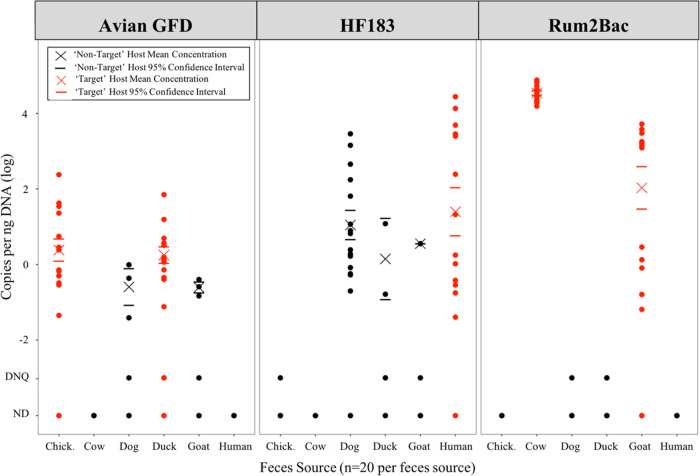
Concentrations of microbial source tracking assay target gene copies per nanogram of DNA extracted. Feces sources analyzed include chicken, cow, dog, duck, goat, and human feces. Target host data points (i.e., chicken and duck data for the avian-specific Avian GFD assay) are represented in red, and the nontarget data points are represented in black. The mean concentration in each source and assay are marked by a cross, with the mean’s 95% confidence interval marked as horizontal lines above and below the mean. Samples where the target was detected but not quantifiable (below 10^1^ copies per μL), and where the target was not detected, are marked at detected but not quantifiable (DNQ) and nondetect (ND), respectively.

All three MST assay gene markers were detected in both target and nontarget host feces (see Table 1). The Avian GFD assay was 82.5% sensitive and 90.0% specific. The HF183 assay was 70.0% sensitive and 78.0% specific. The Rum2Bac assay was 85.0% sensitive and 95.0% specific. The Avian GFD target was detected at quantifiable and nonquantifiable concentrations in dog and goat feces, but at statistically significantly lower (Kruskal–Wallis Dunn’s test, *p* < 0.05) mean concentrations compared to the “target” hosts of chickens and ducks (see Figure 1). The HF183 target was detected in all feces sources but cows, and the mean target concentration in human feces was not statistically significantly different (Kruskal–Wallis Dunn’s test, *p* < 0.05) compared to the nontarget sources of ducks, goats, and dogs. High detection of the HF183 assay in dog feces contributed to a lower specificity of HF183 compared to the other assays. The Rum2Bac target was detected but not quantifiable in the nontarget sources of dog and duck feces.

**Table 1:** Number of Samples (*n* = 20 per Feces Source) by Feces Source Detecting Each Microbial Source Tracking (MST) Assay Using Quantitative Polymerase Chain Reaction^a^

assay	chicken (*n* = 20)	cow (*n* = 20)	dog (*n* = 20)	duck (*n* = 20)	goat (*n* = 20)	human (*n* = 20)	sensitivity	specificity
Avian GFD	*15 (75%)	0 (0%)	4 (20%)	*18 (90%)	4 (20%)	0 (0%)	0.825	0.90
HF183	1 (5%)	0 (0%)	15 (75%)	4 (20%)	2 (10%)	*14 (70%)	0.70	0.78
Rum2Bac	0 (0%)	*20 (100%)	1 (5%)	3 (15%)	*14 (70%)	0 (0%)	0.85	0.95

a The “target” host for each assay (e.g., human feces for HF183) are indicated with an asterisk for identification in the table. The “correct” or “target” hosts are chicken and duck feces for the Avian GFD assay, human for the HF183 assay, and cow and goat for the Rum2Bac assay.

### Enteric Pathogens Detected in Human and Domesticated Animal Feces

**Figure 2:**
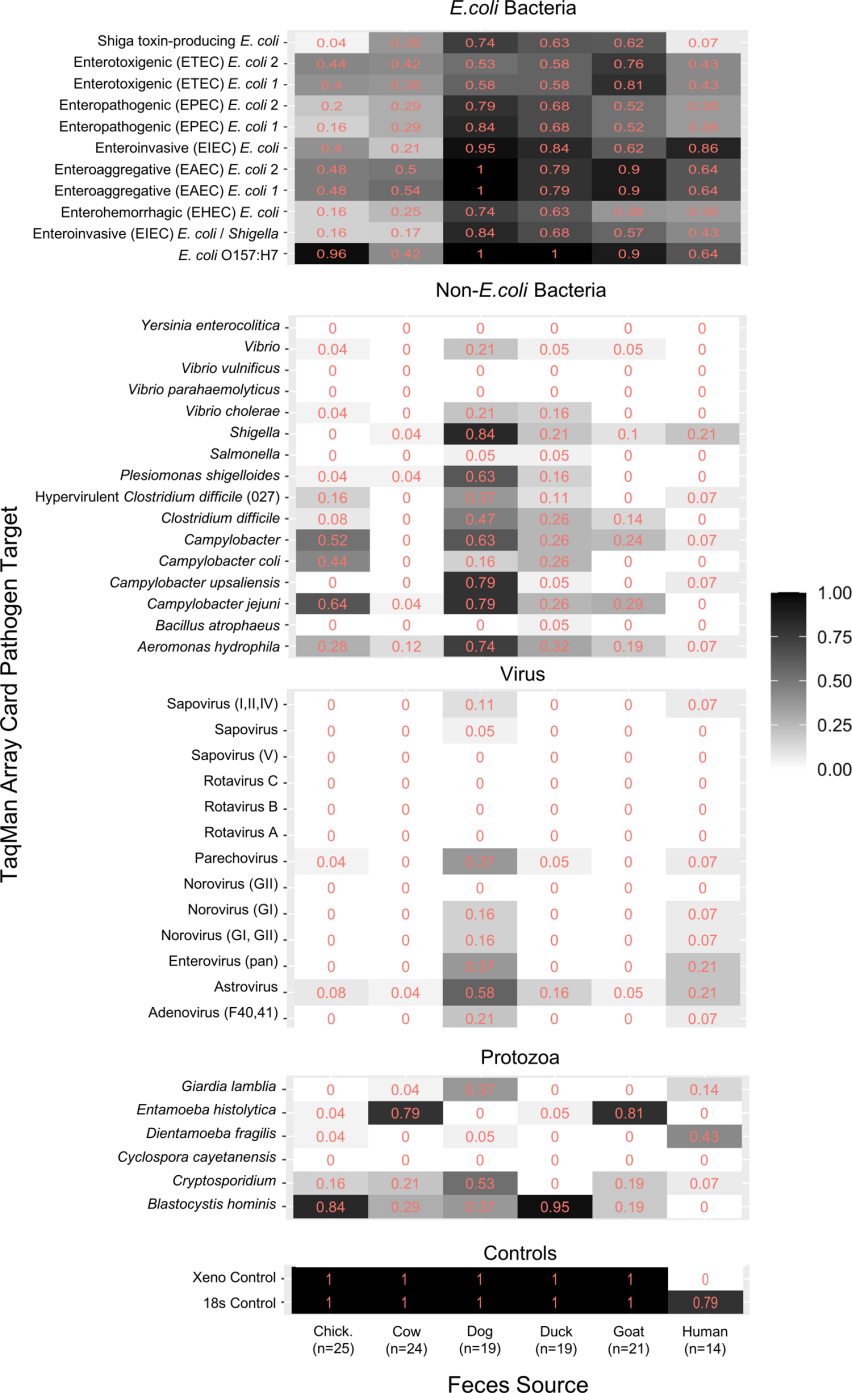
Proportion of samples detecting individual pathogen targets by fecal source. The heatmap represents the proportion of positive samples for each pathogen target, separated by the source of feces analyzed, including chicken, cow, dog, duck, goat, and human feces. The heatmap operates between white cells, representing zero samples from a specific host being positive for an individual pathogen target, to black cells, representing 100% of samples from a specific host being positive for an individual pathogen target. Results are separated by the pathogen type, separating bacterial (*E. coli* and general), viral, protozoan, and control targets.

For the TAC analysis, 25 chicken feces samples, 24 cow feces samples, 19 dog feces samples, 19 duck feces samples, 21 goat feces samples, and 14 human feces samples, were used (see Figure 2). Dog feces contained statistically significantly (Kruskal–Wallis Dunn’s test, *p* < 0.05) the most pathogens (excluding controls, duplicate targets, and targets that include multiple pathogens) on average (range = 7–19, median = 15), followed by duck,^3−15^ goat,^2−10^ human (2–11, 5.5), chicken,^1−10^ and cow^1−12^ feces, which were not significantly different from each other. A Shapiro-Wilk normality test was used to determine the distribution of the number of pathogens detected in each sample and within each feces source type. Each source type fit a normal distribution, except for cow feces.

The first, second, and third most commonly detected pathogens for each source are as follows: *E. coli* O157:H7, *Blastocystis hominis*, and *Campylobacter jejuni* in chicken feces; *Entamoeba histolytica*, enteroaggregative *E. coli*, and *E. coli* O157:H7 in cow feces; *E. coli* O157:H7/enteroaggregative *E. coli*, enteroinvasive *E. coli*, and *Shigella* in dog feces; *E. coli* O157:H7, *B. hominis*, and enteroinvasive *E. coli* in duck feces; *E. coli* O157:H7/enteroaggregative *E. coli*, *E. histolytica*, and enterotoxigenic *E. coli* in goat feces; and enteroinvasive *E. coli*, *E. coli* O157:H7/enteroaggregative *E. coli*, and enterotoxigenic *E. coli*/*Dientamoeba fragilis* in human feces. Pathogens detected in all hosts include: *Aeromonas hydrophila*, astrovirus, *Cryptosporidium*, pathogenic *E. coli* (O157:H7, enteroinvasive, enterohemorrhagic, enteroaggregative, enteropathogenic, enterotoxigenic, and shiga toxin-producing), and *Giardia lamblia*. Any pathogen detected in human feces was also detected in the feces of at least one domesticated animal. Dog feces in particular contained a wide range of pathogens, with many viral targets being detected in at least one individual dog sample. This included viruses typically associated with human infections, and some were observed in higher prevalence in dogs (26% of dog fecal samples contained the adenovirus F40/41 target) compared to humans (7% contained the adenovirus F40/41 target). In fact, the only pathogens detected in human feces using the TAC in this setting that were not detected in at least one dog feces sample were *Bacillus atrophaeus* and *E. histolytica*.

Some pathogens were more likely to be detected in one type of feces (two-proportion *Z*-test, *p* < 0.05), meaning that the detection rate of a pathogen target was statistically significantly higher in one feces group (e.g., dog or ruminant feces) compared to all others. *A hydrophila*, *Campylobacter upsali*, *Plesiomonas shigelloides*, and *Shigella* were statistically correlated with dog feces. *D. fragilis* was statistically correlated with human feces. *E. histolytica* was correlated with ruminant feces, and *B. hominis* was correlated with poultry feces.

### Comparing Multiple Measures of Drinking Water Quality

Indicator *E. coli* was detected in 23% (3/13) of source water samples, with an average of 3 CFU per 100 mL (standard deviation = 3.5), and in 54% (25/46) of stored water samples, with an average concentration of 43 CFU per 100 mL (standard deviation = 99.5) (including one sample that was too numerous to count). The HF183 marker was detected in 7.7% (1/13) of source water and in 2.2% (1/46) of stored water samples, all below the level of quantification. The source water that was positive for HF183 was sampled in duplicate, and each of the duplicate samples detected HF183 below the quantification level. The stored water sample which contained the HF183 marker was not collected from the source water that tested positive. There was no detection of the Avian GFD or Rum2Bac markers in any of the water samples or associated blanks. However, being below the 0.8 sensitivity and specificity threshold, detection of HF183 in water is inconclusive for human feces.

According to the TAC results, stored drinking water exhibited higher contamination, on average, compared to source drinking water, both in nucleic acid present (represented by the 18s control, 31% detection in source vs 61% in stored) and in average number of pathogens detected (0.31 pathogens on average for source waters and 0.63 pathogens on average for stored waters). *Vibrio* and *B. hominis* were detected in 7.7% (1/13) and 15% (2/13), respectively, of source water samples, but were not detected in stored waters. *A. hydrophila* was detected in 23% (3/13) of source waters and 15% of stored (7/31) waters. *Cryptosporidium* was detected in 19% (6/31) of stored waters, but was not detected in source water samples. Of stored water samples analyzed, up to 9.7% (3/31) contained enteroaggregative *E. coli*, 9.7% (3/31) contained enterotoxigenic *E. coli*, and 3.2% (1/31) contained enteropathogenic *E. coli*. Pathogenic *E. coli* gene targets were not detected in source waters.

There were no statistically significant associations between either the source of water or the storage container used and *E. coli* contamination, HF183 detection, or any detected pathogens. There were also no statistically significant associations between source vs stored water and *E. coli* contamination, HF183 detection, or any detected pathogens. However, the association between stored water and the presence of *E. coli* was close to significant, with a *p*-value of 0.06. There were also no statistically significant associations between the presence of *E. coli* contamination and HF183 detection or any detected pathogens. The stored water sample and the source water sample which contained the HF183 marker both contained *E. coli*. However, *Aeromonas hydrophilia* was detected in 1 source water and 3 stored water samples which did not contain *E. coli*. Additionally, *B. hominis* was detected in one *E. coli*-free source water sample, and *Cryptosporidium* and enterotoxigenic *E. coli* were each detected in one stored water sample.

All inhibition and quality control results are available in the Supporting Information.

## Discussion

Our study used gastrointestinal TaqMan Array Cards to investigate the presence of enteric pathogens in both domesticated animal and human feces in a dense, low-income area in Dagoretti South, Nairobi, Kenya. We detected multiple enteric pathogens in the feces of various domesticated animals (including a maximum of 24 different positive pathogen targets in a 0.25-g sample of dog feces), identifying potential human exposure to pathogens associated with animal feces in the study setting. The impact of domesticated animal feces on water, sanitation, and hygiene conditions is rarely targeted in intervention and monitoring campaigns,^13,40^ and we have potentially identified canine feces as an important source of human pathogens in this area. This compliments a United States-based study,^41^ which also identified the threat of canine zoonotic pathogens. Specifically, we detected the highest average number of pathogens in dog feces (15.5 pathogens on average per sample). Dog feces also contained the largest diversity of pathogens, with only two pathogens detected in feces in this setting not being detected in dogs. Exposure to dog feces has been associated with soil-transmitted helminth seropositivity,^9,22^ as well as child infection of *C. jejuni* and enteropathogenic *E. coli* (EPEC),^9^ which we detected in 79 and 84% of dog fecal samples, respectively. Conan et al. (2017)^42^ investigated the animal-related factors and pathogen infections associated with moderate or severe diarrhea in children in Kenya. They detected *Campylobacter* (both *C. jejuni* and *C. coli*), nontyphoidal *Salmonella*, enteroaggregative *E. coli*, *Giardia*, and *Cryptosporidium* in domestic dog feces, and identified *Giardia* and *Salmonella* in the feces of both dogs and children under 5 years experiencing moderate-to-severe diarrhea within the same household. Harvey et al. (2020)^43^ identified overlapping infections of *Giardia* and *Cryptosporidium* between children and dogs in Brazil and observed frequent contact between dogs and children as potentially promoting zoonotic pathogen transmission. Penakalapati et al. (2017)^9^ suggest that improving animal containment and feces management as target areas for reducing the risk of exposure to animal (including canine) feces, but that further research is needed. Prendergast et al. (2019)^40^ suggest including animal feces management as a core tenant to WASH management, and we contend that dog feces should be included in the consideration of hazardous waste among other traditional domesticated animals. Understanding exposure to dog feces is a necessary first step to determining if proper canine feces management interventions have potential to reduce negative health outcomes.

Our TAC analysis detected *Campylobacter* in 65 and 25% of chicken and duck fecal samples, respectively, and in 9% of human fecal samples, which suggests the possibility for poultry-to-human *Campylobacter* transmission. Zambrano et al. (2014)^20^ conducted a systematic review and meta-analysis which identified a relationship between exposure to domestic poultry and subsequent infection with *Campylobacter*. *Cryptosporidium* in the stool of children has been associated with household presence of chickens in Cambodia,^44^ and we observed similar rates of *Cryptosporidium* detection between chicken feces (13%) and human feces (9%) in Kenya. In 40% of cow and 67% of goat feces, respectively, we detected shiga toxin-producing *E. coli* (STEC); a pathogen that has been associated with millions of acute illnesses annually, and exposure to ruminant feces is considered critical to the burden of disease associated with STEC.^21^

We found that all pathogens detected in human feces were also detected in domesticated animal feces, highlighting potential for animal-human transmission of pathogens. In both humans and domesticated animals, we detected: enteric viruses such as adenovirus, astrovirus, enterovirus, norovirus, parechovirus, and sapovirus; enterotoxigenic, enteropathogenic, enteroaggregative, enteroinvasive, shiga toxin-producing, and O157:H7-type *E. coli*; and enteric protozoan pathogens such as *Cryptosporidium* and *E. histolytica*. There is evidence that humans can share elements of their microbiome with animals they are in close contact with, such as pets (i.e., dogs),^45^ further highlighting how microorganism transmission may occur between humans and their domesticated animals. However, it is unclear in which direction this pathogen exchange between animals and humans is occurring, from animal to human, vice versa, or in both directions. In addition, humans and one animal host, such as dogs, may both be exposed to a pathogen sourced from a different animal host, such as cows. Further temporal investigation of pathogen mobilization and transmission throughout environmental reservoirs is needed to properly evaluate the level of animal-to-human pathogen exposures. Genetic sequencing could also prove useful for understanding the specific characteristics of pathogen strains in different hosts.^46^

There is also substantial lack of knowledge surrounding which pathogens are consistently prevalent in the feces of certain hosts, and our results do not always support existing literature. *Campylobacter* is considered common in poultry and cattle feces.^21^ While we detected multiple species (*C. coli*, *C. upsaliensis*, *C. jejuni*) in various hosts, we did not detect *Campylobacter* in cow feces. Norovirus has been identified as a human-hosted pathogen,^6,21^ while we detected it in both human and dog feces. *Shigella* has been claimed to be hosted by humans and related primates,^6,21,47^ however we detected *Shigella* in all analyzed hosts except chickens, including in 85% of dog fecal samples. Adenovirus F40/41 has been considered human-specific,^21,48^ however we detected it in higher rates in dog feces compared to human feces. Human norovirus (i.e., strains typically associated with human infections) has been detected in domesticated dogs in contact with infected humans,^49^ human adenovirus has been detected in dog feces,^50^ and *Shigella* has been isolated in asymptomatic dogs.^51^ However, it is uncertain whether molecular targets isolated in unexpected hosts indicate pathogenicity or transmissibility to humans, but the potential exists. These discrepancies highlight the gap in knowledge surrounding host–pathogen relationships in various domesticated animals and humans, and in different environmental contexts. This is valuable information for conducting accurate risk assessments and disrupting pathways by which humans are exposed to pathogens. Further research is needed on the temporal and spatial mobilization of pathogens, and the pathogen profile of animal and human hosts in order to fully understand and mitigate zoonotic disease transmission.

Our study also used MST for human, avian, and ruminant feces to identify the source of fecal contamination in both source and stored drinking waters in this urban area of Kenya. As each assay in this study was detected but not quantifiable in some samples, we primarily report the sensitivity and specificity using a common binary threshold.^27^ Using this metric, we determined the Rum2bac and Avian GFD assays to be sensitive and specific in this setting to their target hosts, ruminant and avian animals, respectively. However, the HF183 assay did not reach this threshold for either sensitivity or specificity, meaning that HF183 markers detected in drinking water are not conclusive for human fecal contamination and may contain type 1 and/or type 2 errors. Hamzah et al. (2020)^29^ investigated these same markers (among others) in a rural setting in Kenya, finding all sensitive under the 0.8 threshold, but finding HF183, Rum2Bac, and Avian GFD not sufficiently specific under the binary criterion. Boehm et al. (2013)^27^ evaluated the performance of nine human-specific MST markers in multiple United States-based laboratories, finding the HF183 TaqMan assay sensitive but not specific using this binary metric. These assay performance results are relatively similar to studies using these assays in other settings, but assay performance vary across various context (e.g., geographies, urban vs rural context)^16,27,38,39^ and validating assays in specific contexts should be done before using them to evaluate environmental samples. As a particular challenge, we detected high levels of cross-detection of the HF183 marker in dog feces. We also found cross-detection of the Avian GFD and Rum2Bac markers in dog feces. Human MST assays have been observed to cross-react with dog feces in previous studies,^30^ and our TAC analysis suggests that their pathogen profiles contain substantial overlap as well. Dogs often live in close proximity to other animals, including humans, and have been observed to consume the feces of those other animals.^52^ Overlapping omnivorous diets (e.g., households feeding dogs food scraps/waste) could also promote similar gut microbiomes.^53^ These canine characteristics could contribute to the cross-detection of nondog MST assays and pathogens, and new assays are needed to effectively differentiate between dog feces and other hosts. However, in this context, we suggest that Rum2Bac is effective for identifying ruminant fecal contamination, Avian GFD is acceptable for identifying avian fecal contamination, and HF183 is not validated in this setting.

There are limitations associated with the methods presented in this study. Cost and logistic considerations (particularly the high per-sample cost of TACs) resulted in a small sample size, as we elected to prioritize robust analysis (e.g., *E. coli*, MST targets, and pathogen analyses) on the samples collected, which reduced the statistical power of our analysis and the generalizability of our results to other contexts. Viral targets were not analyzed in drinking water samples as the filtration and extraction methods with these samples only isolated DNA. Molecular methods, such as MST and TAC, amplify gene sequences, but they do not shed light on the viability of detected genes or organisms. These methods do not provide information to determine if the nucleic acid detected was extracted from inactivated cells and viable cells that would be pathogenic.^54^ There are also limitations associated with certain molecular targets. *E. coli* O157:H7 has been previously identified using a gene unique to O157:H7, *rfbE.*(55,56) However, there is evidence that the strain may not be toxigenic unless the *rfbE* gene is accompanied by a shigatoxin (*stx*) gene.^55,57^ Culturing organisms is still required to make certain that nucleic acid detected is from viable organisms, and careful selection of gene targets is needed to ensure pathogen targets properly represent pathogenic organisms. Our samples were collected as 0.25 g of feces and 100 mL of water, which partially drives the detection limit of the assays. The small reaction volume used in the TAC, 1.5 μL per target, has been attributed to a lower sensitivity compared to qPCR techniques.^33^ However, our preamplification step was included to increase the sensitivity of the TAC to detect low concentration targets.^32,58^ Additionally, collecting deposited feces from the ground introduces uncertainty surrounding the age of feces samples. While trained personnel were employed to identify and prioritize freshly deposited feces, aging feces could lead to degradation of contained organisms and genes. Given these limitations, there could be false negatives represented in our analysis. We also lack the robust temporal sampling scheme of the feces of humans and their associated domesticated animals that would allow for identifying the direction of animal-human pathogen transmission, therefore, we do not have the ability to estimate whether animal-to-human or human-to-animal transmission is the primary pathway. The preamplification step also introduces additional uncertainty into quantifying the starting concentration of pathogen targets in the samples, so the data is reported as binary instead of quantitative. Other studies have made quantitative estimates with TAC results using known concentrations of targets and standard curves, which is useful information to apply to risk assessments.^31,33^

Our study used multiple methods for evaluating the contamination of source and stored drinking water in the Dagoretti South constituency of Nairobi, Kenya, including traditional indicator *E. coli*, host-specific microbial source tracking, and pathogen-detecting TaqMan Array Cards. While the MST assays did not yield useful data, the *E. coli* and TAC data suggest contamination in both the source and stored drinking water in this area. While the connection between animal feces runoff and surface waters is clear, there is also evidence in the literature of animal feces contaminating groundwater drinking water sources as well.^9^ Using TACs, we identified *A. hydrophilia* and *B. hominis* in source drinking water, which were also present in dog feces and poultry feces, respectively, in this setting. Using these tools in a continuous, temporal sampling campaign of water sources, animal feces, and human feces would also inform the directionality of animal-human pathogen transmission. Failure to manage domesticated animal feces could result in contaminated drinking water and reduced benefits from traditional WASH improvements,^13−15,40^ and this study suggests that TACs can be a powerful tool for characterizing the risks associated with animal feces and environmental transmission pathways, such as drinking water.

There is evidence that coliform indicators, such as *E. coli*, may not correlate with enteric viruses and protozoan parasites.^26^ We detected two protozoan pathogens (*B. hominis* and *Cryptosporidium*) in drinking water samples in which we did not detect indicator *E. coli*. We also detected three bacterial pathogens in samples which did not contain indicator *E. coli*, including pathogenic enterotoxigenic *E. coli*. This could partially be explained by the TAC amplifying nucleic acid from both viable and nonviable cells, while the indicator *E. coli* was cultured, detecting only viable cells. TACs have the capacity to expand surveillance of a wide range of pathogens, and have been used in this study and others^33,58^ across multiple animal hosts and environmental reservoirs. Molecular methods have been used for surveillance of pathogens in municipal wastewater,^59^ and others^33,60^ have successfully used TACs to detect pathogen targets in wastewater. Application-specific TACs could be successfully used for monitoring animal-specific diseases in agricultural settings, and for broad, community wide disease surveillance via wastewater monitoring.

This study highlights issues surrounding the microbial quality of drinking water, potential sources of fecal contamination in water, and health-hazards of domesticated animal and human feces in Nairobi, Kenya. We provide insight on pathogen-host relationships in this setting, which informs our understanding of the zoonotic transmission potential of different pathogens. This information can be used by residents and human health researchers to make more informed decisions regarding managing and preventing potential pathogen exposures associated with certain domesticated animal hosts. We also demonstrate the utility of TaqMan Array Card methods for evaluating environmental contamination and hazards posed to humans, and identified exposures and risk associated with dog feces as specifically warranting further investigation. While using traditional fecal indicator organisms indicates the presence of feces in environmental samples, molecular methods can be powerful tools for identifying risks and identifying transmission pathways of pathogens to humans, and pairing TAC and culture-based methods could help address limitations in both strategies. Adding setting-validated MST targets to TACs is also feasible, and would allow for the simultaneous detection of enteric pathogen genes in the environment and the potential source of fecal contamination in an environmental sample. This would allow for robust risk characterization to be achieved and enhance capabilities for a One Health approach for effective prevention and management of disease outbreaks.

## Supporting Information Available

PCR inhibition, qPCR assays-methods, TaqMan array cards-methods, inhibition and quality control-results (PDF)
GI target analysis for NCSU TaqMan gastrointestinal Card v.3 (XLSX)
