# Supplementary material for: Enteric Pathogens in Humans, Domesticated Animals, and Drinking Water in a Low-Income Urban Area of Nairobi, Kenya
Source: Environ Sci Technol. 2024 Nov 26;58(49):21839–49. doi: 10.1021/acs.est.4c10041 (PMC11636211; doi:10.1021/acs.est.4c10041)
Supplement: Supplementary file 1 — es4c10041_si_001.pdf [file es4c10041_si_001.pdf]

Title: Enteric Pathogens in Humans, Domesticated Animals, and Drinking Water in a Low-Income Urban Area of Nairobi, Kenya

Authorship: Sean W. Daly[1], Benard Chieng[2], Sylvie Araka[2], John Mboya[2,3], Christine Imali[4], Jenna M. Swarthout[5], Sammy M. Njenga[2], Amy J. Pickering[3,6], Angela R. Harris[1*]

[1]North Carolina State University, Department of Civil, Construction, and Environmental Engineering, Fitts-Woolard Hall, 915 Partners Way, Rm 3250, Raleigh, NC 27695, United States
[2]Kenya Medical Research Institute, Nairobi 00100, Kenya
[3] University of California, Berkeley, Department of Civil and Environmental Engineering, Berkeley, California 94720, United States
[4] Innovations for Poverty Action, Nairobi 00100, Kenya
[5] Tufts University, Civil and Environmental Engineering, Medford, Massachusetts 02155, United States
[6] Chan Zuckerberg Biohub, San Francisco, California 94158, United States

*Email: aharris5@ncsu.edu

Number of pages: 9

Number of figures: 3

Number of tables: 13

PCR Inhibition

qPCR Assays-Methods

Each feces source's potential for PCR inhibition was evaluated using established 'dilute and spike' methods (Harris et al. 2016). Four samples from each source (20% of total) were decimally diluted 1:10 and 1:100. The undiluted, 1:10 dilution, and 1:100 dilution of each sample were run, both with and without a 10^3 copies per µL spike of standard (spiked after dilution). The HF183 and Avian GFD assays were used to analyze inhibition for the TaqMan and SYBR assays, as HF183 and Rum2Bac use the same TaqMan reagents. To determine if a sample contained PCR inhibitors, the Ct values for a spiked sample and its unspiked counterpart were compared (i.e., spiked 1:10 diluted sample vs. unspiked 1:10 diluted sample). If a spiked sample had a Ct value over 3 cycles higher than its unspiked counterpart's Ct value, the sample was considered inhibited (Harris et al. 2016). For the TaqMan and SYBR assays, sample types that did not exhibit inhibition were analyzed without dilution, while those that did exhibit inhibition were diluted 1:10 prior to analysis.

TaqMan Array Cards-Methods

Samples were considered inhibited if the Xeno extraction control target within samples amplified with a Ct value of 4 or greater than the Xeno control Ct value of the sample's associated extraction blank (Baker et al. 2018). Any samples that exhibited inhibition were diluted 1:10 prior to preamplification. If inhibition was identified and was not resolved with a 1:10 dilution, the results were excluded from the final TAC analysis. If the 1:10 dilution did resolve the inhibition, then the positive detections from both the undiluted and 1:10 diluted samples were included.

As the Xeno extraction control was not spiked into human fecal samples and drinking water samples prior to analysis, inhibition could not be evaluated this way. Instead, a Xeno control qPCR assay was obtained (Thermo Fisher Scientific,Waltham, MA) and the TAC's PCR reaction was replicated to evaluate the potential of the human feces samples and a subset of the water samples for inhibiting the amplification of the Xeno control target. The reaction mixtures and thermocycling parameters for this inhibition test are seen in Tables SI 12-13. First, a preamplification reaction was prepared (see Table SI5), with Xeno control added to achieve a concentration of 2×10^4 copies per µL, the volume that is contained in nucleic acid extracted with Xeno added prior to extraction. These samples were then preamplified (see Table SI9), and the preamplified samples were added to the qPCR reaction mixture seen in Table SI12 for evaluating the potential inhibition of the human fecal and drinking water samples.

Inhibition & Quality Control-Results

All sample types were uninhibited with the HF183 assay at the undiluted level, therefore, feces samples were analyzed for the TaqMan HF183 and Rum2Bac assays without dilution. For the Avian GFD assay, all sample types were inhibited at the undiluted level for all feces sources except for cow feces, which did not exhibit inhibition. Therefore, 1:10 dilutions of feces samples were used for the Avian GFD assay analysis, with the exception of cow feces being run undiluted. A subset of drinking water samples—four stored water and four source water samples—were also analyzed for inhibition, with no evidence of inhibition being found for the MST assays.

Seven (5.6%) of the feces samples included in the TAC analysis exhibited inhibition. A 1:10 dilution prior to preamplification resolved the inhibition in 5 of the 7 inhibited samples. The

two samples (one dog and one duck sample) that still exhibited inhibition at the 1:10 dilution were excluded from the final analysis. Ten (22% of total) stored water samples and 4 (31% of total) source water samples were evaluated for inhibition with the TACs. Two stored water and 1 source water samples were spiked with the Xeno control prior to extraction, and none exhibited inhibition with the TACs. Eight stored water samples and 3 source water samples were analyzed with the Xeno qPCR assay, which also did not exhibit any inhibition.

There were also no significant differences observed between the 5 samples that did not contain sodium thiosulfate and the 54 that did. All results presented are associated with uncontaminated extraction, field, and lab blanks, and no detected PCR inhibition.

Reagent	Initial Concentration	Final Concentration	Volume Added to Reaction (µL)
TaqMan Environmental Master Mix 2.0	2X	1X	15
HF183 Primer Probe-Mixture			3
7.5% BSA	7.5 mg/mL	0.2 mg/mL	0.8
Nuclease-Free Water			9.2
Template DNA			2
Total			30

Table SI1: HF183 qPCR reaction mixture.

Reagent	Initial Concentration	Final Concentration	Volume Added to Reaction (µL)
TaqMan Environmental Master Mix 2.0	2X	1X	15
Rum2Bac Primer-Probe Mixture			3
7.5% BSA	7.5 mg/mL	0.4 mg/mL	1.6
Nuclease-Free Water			8.4
Template DNA			2
Total			30

Table SI2: Rum2Bac qPCR reaction mixture.

Reagent	Initial Concentration	Final Concentration	Volume Added to Reaction (µL)
PowerTrack SYBR Green Master Mix	2X	1X	10
Yellow Tracker Dye			0.5
Avian GFD Primer-Probe Mixture			1
7.5% BSA	7.5 mg/mL	0.04 mg/mL	0.107
Nuclease-Free Water			6.393
Template DNA			2
Total			20

Table SI3 Avian GFD qPCR reaction mixture.

**Reagent**	**HF183 (µL for 1 reaction)**	**Rum2Bac (µL for 1 reaction)**	**Avian GFD (µL for 1 reaction)**
Forward Primer	1	0.2	0.3
Reverse Primer	1	0.2	0.3
Probe	0.08	0.2	N/A
Nuclease-Free Water	0.92	2.4	0.4
Total	3	3	1

Table SI4: All microbial source tracking qPCR assay primer-probe reaction mixtures.

**Reagent**	**Volume per Reaction (µL)**
TaqPath 1-Step RT-qPCR Master Mix, CG	2.5
TrueMark GastroIntestinal Panel 3.0 PreAmp Primer Pool	2.5
Sample DNA	5
Total	10

Table SI5: TaqMan Array Card preamplification reaction mixture.

**Reagent**	**Volume per Sample (µL)**
1:10 Diluted Preamplified Sample DNA	20
TaqMan Fast Advanced Master Mix, no UNG	50
Nuclease-Free Water	30
Total	100

Table SI6: TaqMan Array Card reaction mixture.

**Step**	**Temperature (ºC)**	**Time**	**Cycles**
Hold	95	10:00	1
Denature	95	00:15	40
Anneal/Extend	60	01:00	

Table SI7: Thermocycling parameters for TaqMan Environmental Master Mix 2.0, used for the HF183 and Rum2Bac assays.

**Stage**	**Step**	**Temperature (ºC)**	**Ramp Rate (ºC/second)**	**Time**	**Cycles**
PCR	Enzyme Activation	95	N/A	02:00	1
	Denature	95	N/A	00:05	40
	Anneal/Extend	60	N/A	00:30	

Melt Curve	1	95	1.99	00:15	1
	2	60	1.77	01:00	1
	3 (Dissociation)	95	0.075	00:15	1

Table SI8: Thermocycling parameters for PowerTrack SYBR Green Master Mix, used for the Avian GFD assay.

**Step**	**Temperature (ºC)**	**Time**	**Cycles**
UNG Incubation	25	02:00	1
Reverse Transcription	50	30:00	1
Activation	95	02:00	1
Denature	95	00:15	14
Anneal/Extend	60	02:00	
Inactivation	99.9	10:00	1
Hold	4	N/A	N/A

Table SI9: Thermocycling parameters for TaqMan Array Card preamplification step.

**Step**	**Temperature (ºC)**	**Time**	**Cycles**
Activation	95	10:00	1
Denature	95	00:03	40
Anneal/Extend	60	00:30	

Table SI10: Thermocycling parameters for TaqMan Array Card analysis.

**General Pathogen Group**	**Individual TaqMan Array Card Targets**
Internal Controls	18s Control (Amplification Control)<br>Xeno Control (Extraction Control)
Adenovirus	Adenovirus F40/41
Aermonas hydrophila	
Astrovirus	
Bacillus atrophaeus	
Blastocystis hominis	

Campylobacter	*Campylobacter*<br>*Campylobacter coli*<br>*Campylobacter jejuni*<br>*Campylobacter upsaliensis*
Clostridium difficile	*Clostridium difficile*<br>Hypervirulent *Clostridium difficile* (027)
Cryptosporidium	
Cyclospora cayetanensis	
Dientamoeba fragilis	
Escherichia coli	Enteroaggregative (EAEC) *E. coli* (dup)<br>Enterohemorrhagic (EHEC) *E. coli*<br>Enteroinvasive (EIEC) *E. coli* / *Shigella*<br>Enteropathogenic (EPEC) *E. coli* (dup)<br>Enterotoxigenic (ETEC) *E. coli* (dup)<br>*E. coli* O157:H7<br>Shiga toxin-producing *E. coli*
Giardia lamblia	
Norovirus	Norovirus GI<br>Norovirus GII<br>Norovirus GI, GII
Parechovirus	
Plesiomonas shigelloides	
Rotavirus	Rotavirus A<br>Rotavirus B<br>Rotavirus C
Sapovirus	Sapovirus<br>Sapovirus I, II, IV<br>Sapovirus V

Shigella	*Shigella*<br>Enteroinvasive (EIEC) *E. coli* / *Shigella*
Vibrio	*Vibrio cholerae*<br>*Vibrio parahaemolyticus*<br>*Vibrio vulnificus*
Yersinia enterocolitica	

Table SI11: All TaqMan Array Card (TAC) PCR targets for detecting multiple pathogens simultaneously. Targets that were run in duplicate on the card are denoted with “(dup).” As the Gastrointestinal Trial Card, Version 3 TAC platform is a proprietary product of ThermoFisher, specific gene targets and associated sequences are not available for publication.

**Reagent**	**Volume per Sample (µL)**
1:10 Diluted Preamplified Sample DNA	4
TaqMan Fast Advanced Master Mix, no UNG	10
TaqMan Assay (20X)	1
Nuclease-Free Water	5
Total	20

Table SI12: Reaction mixture for qPCR inhibition experiment for human fecal and drinking water samples.

<table>
<tr><th>Step</th><th>Temperature (ºC)</th><th>Time</th><th>Cycles</th></tr>
<tr><td>Activation</td><td>95</td><td>00:20</td><td>1</td></tr>
<tr><td>Denature</td><td>95</td><td>00:01</td><td rowspan="2">40</td></tr>
<tr><td>Anneal/Extend</td><td>60</td><td>00:20</td></tr>
</table>

Table SI13: Thermocycling parameters for qPCR inhibition experiment for human fecal and drinking water samples.

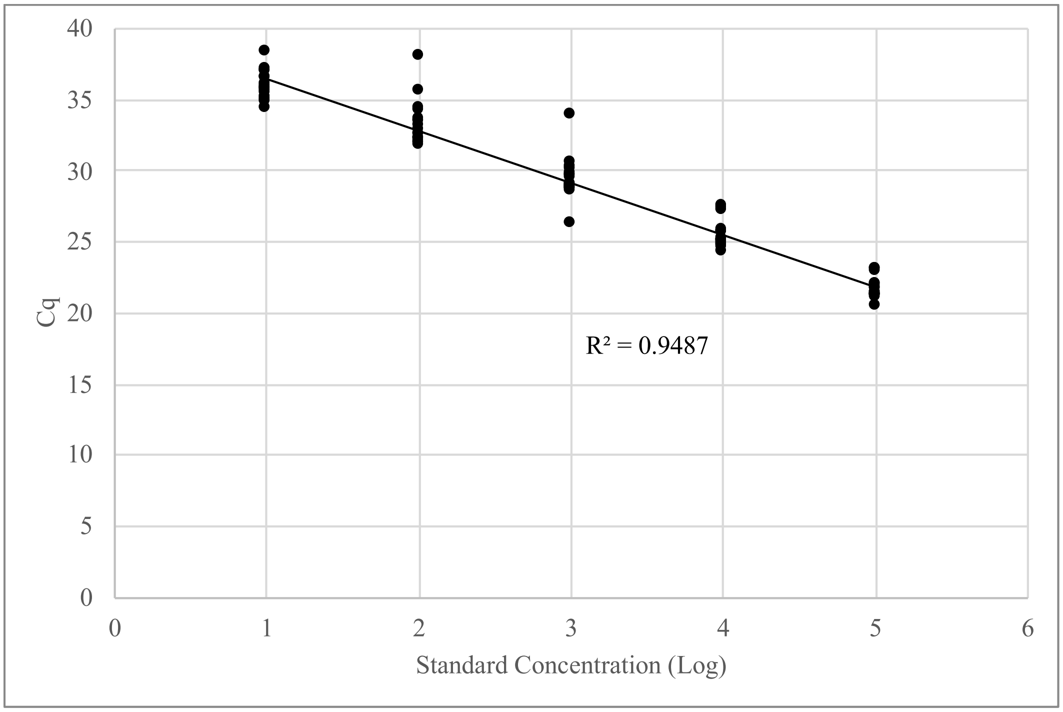

Figure SI1: Master standard curve for the HF183 qPCR assay. Each qPCR plate was run with a 5-point standard curve, and concentration estimates for all samples were made based on the standard curve run on the same plate. All individual standard curves had trendline R^2 values above 0.95, and PCR efficiency estimates between 90-110%.

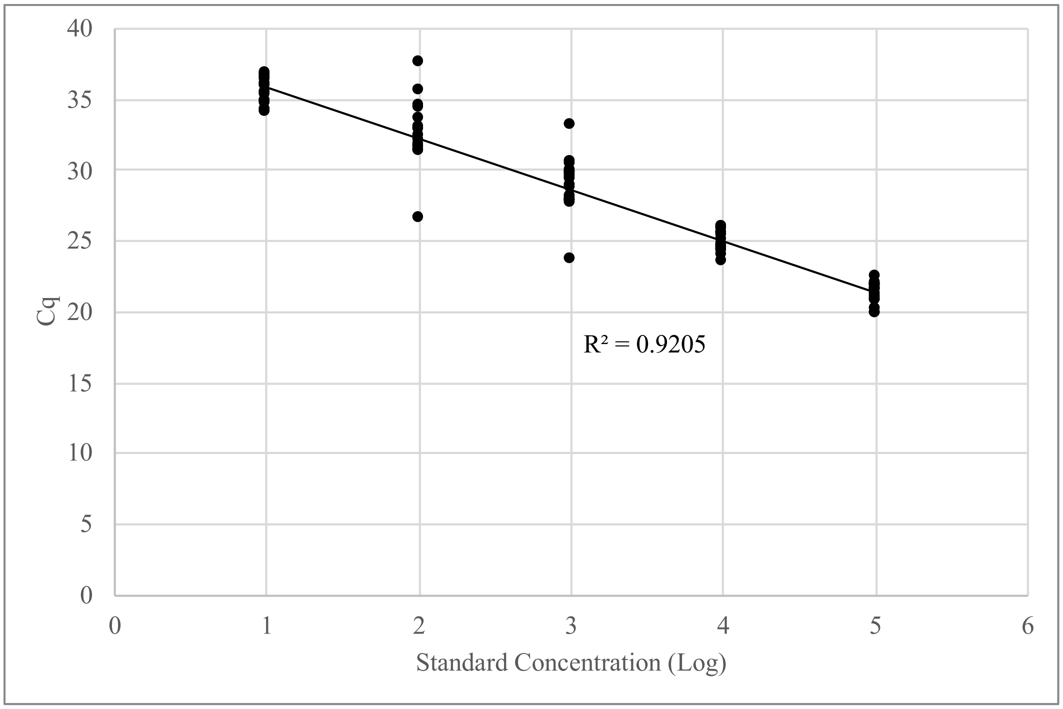

Figure SI2: Master standard curve for the Rum2Bac qPCR assay. Each qPCR plate was run with a 5-point standard curve, and concentration estimates for all samples were made based on the

standard curve run on the same plate. All individual standard curves had trendline R^2 values above 0.95, and PCR efficiency estimates between 90-110%.

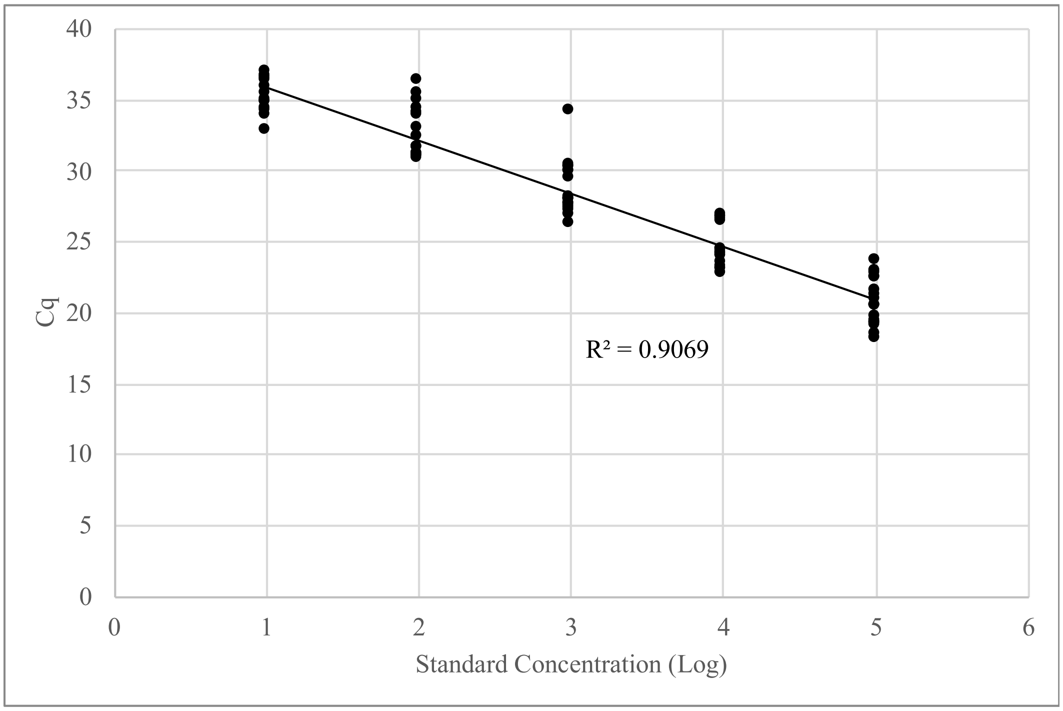

Figure SI3: Master standard curve for the Avian GFD qPCR assay. Each qPCR plate was run with a 5-point standard curve, and concentration estimates for all samples were made based on the standard curve run on the same plate. All individual standard curves had trendline R^2 values above 0.95, and PCR efficiency estimates between 90-110%.

References

Baker, K. K.; Senesac, R.; Sewell, D.; Sen Gupta, A.; Cumming, O.; Mumma, J. Fecal fingerprints of enteric pathogen contamination in public environments of Kisumu, Kenya, associated with human sanitation conditions and domestic animals. *Environmental Science & Technology*, **2018**, *52*(18), 10263-10274.

Harris, A. R.; Pickering, A. J.; Harris, M.; Doza, S.; Islam, M. S.; Unicomb, L.; Luby, S.; Davis, J.; Boehm, A. B. Ruminants contribute fecal contamination to the urban household environment in Dhaka, Bangladesh. *Environmental Science & Technology*, **2016**, *50*(9), 4642-4649.
